# Enhanced anomalous magnetization in carbonyl iron by Ni^+^ ion beam irradiation

**DOI:** 10.1038/s41598-021-99673-3

**Published:** 2021-10-11

**Authors:** Jun Kue Park, Hye Min Jang, Won-Je Cho, Chorong Kim, Jaekwon Suk, Dong-Seok Kim, Jae Sang Lee

**Affiliations:** Korea Multi-purpose Accelerator Complex, Korea Atomic Energy Research Institute, Gyeongju, 38180 Korea

**Keywords:** Ferromagnetism, Magnetic properties and materials, Magnetic materials

## Abstract

We investigate the magnetic properties in carbonyl iron (CI) particles before and after Ni$^{+}$ and H$^{+}$ ion beam irradiation. Upon increasing temperatures, the saturation magnetization ($M_{\text {s}}$) in hysteresis loops exhibits an anomalous increase at a high temperature for the unirradiated and the Ni$^{+}$-beam-irradiated samples, unlike in H$^{+}$-beam-irradiated sample. Moreover, the magnetization values at low and high temperatures are more intense after Ni$^{+}$ beam irradiation, whereas after H$^{+}$ beam irradiation those are remarkably suppressed. Hematite ($\alpha$-Fe$_{2}$O$_{3}$) phase introduced on the surface of our CI particles undergoes the Morin transition that was observed in our magnetization-temperature curves. The Morin transition causing canted antiferromagnetism above the Morin temperature was found in the unirradiated and Ni$^{+}$-beam-irradiated samples, but not in H$^{+}$-beam-irradiated sample. It is thus revealed that the CI particles undergoing the Morin transition cause the anomalous increase in $M_{\text {s}}$. We may suggest that Ni$^{+}$ ion beam increases uncompensated surface spins on the CI particles resulting in a more steep Morin transition and the intensified $M_{\text {s}}$. Ion-beam irradiation may thus be a good tool for controlling the magnetic properties of CI particles, tailoring our work for future applications.

## Introduction

Carbonyl iron (CI) powders are well known as microwave absorption materials due to their superior electric conductivity, high saturation magnetization, high Curie-temperature, and wide absorption bandwidth of 2–18 GHz^1–8^. In particular, a relatively high density and a high filling content make them more proper in electromagnetic wave absorption materials. From a point of view in magnetic properties, CI powders can be applied to magnetorheological suspensions where their favorable magnetic properties change Newtonian or pseudoplastic fluids in the absence of magnetic field to viscoplastic solid in the presence of magnetic field, creating chain-like structures along a direction of the field^4,5^. Their superior controllability of the viscosity in the systems may thus successfully be exploited in industrial applications.

Although there are many studies to investigate the electromagnetic wave absorption properties of CI powder^1–4,7,8^, a comprehensive study for the magnetic properties of CI powders has not been reported so far. Moreover, few studies reported on the understanding of the properties resulting from doping of transition metal ions, in particular. Meanwhile, Pan et al. previously reported that the morphology of the CI particles such as spherical and flaky shapes may also affect the magnetic properties and microwave absorption properties, due to the different magnetic loss mechanisms depending on the particle shape^3^. Particle size is also one of the main factors in changing magnetic properties^3,4,6,9,10^. Furthermore, oxidation process in CI particles may affect magnetization properties in which the saturation magnetization ($M_{\text {s}}$) shows a decrease with oxidation, leading to the formation of maghemite (Fe$_{2}$O$_{3}$)^5^. A decrease of $M_{\text {s}}$ and an increase of the saturation field may arise from a decrease of the Ruderman–Kittel–Kasuya–Yosida (RKKY) interactions. The CI particles may easily be exposed to air during handling and storage, thus giving rise to any Fe$_{2}$O$_{3}$ phase on the surface of the sample^5^. Among iron oxide materials, hematite ($\alpha$-Fe$_{2}$O$_{3}$), an *n*-type semiconductor, is known as the most stable iron oxide under ambient condition^11^. It has many application fields such as catalysts, gas sensors, solar cells, and so on due to its environmentally friendly characteristics.

Unlike in CI exhibiting typical ferromagnetic hysteresis loops^5,7,12,13^, hematite undergoes an anomalous magnetic transition, so-called Morin (spin reorientation) transition due to its antiferromagnetic properties^11,14–19^. It is a well known antiferromagnetic insulator with antiparallel sublattice that above the Morin temperature ($T_{\text {M}}$ = 260 K) a small sublattice canting takes place due to its internal antisymmetric superexchange, Dzyaloshinskii–Moriya (DM), interaction. In this paper, we have investigated the magnetic behaviors of CI particles before and after Ni$^{+}$- and H$^{+}$-beam irradiation. Here, we observed an anomalous increase in $M_{\text {s}}$ at elevated temperature for the unirradiated sample, and an intensified $M_{\text {s}}$ was observed after Ni$^{+}$-ion beam irradiation, whereas beam irradiation with H$^{+}$ remarkably decreases the $M_{\text {s}}$ at low and high temperatures, and leads to no anomalous increase in $M_{\text {s}}$ at a high temperature. We display the results of dc magnetic properties with respect to temperature and magnetic field for the samples before and after the beam irradiation, demonstrating a presence of the Morin transition causing associated anomalous magnetic behavior.

**Figure 1:**
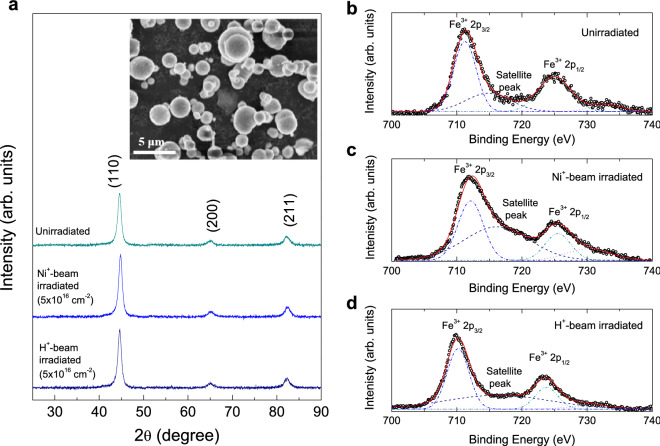
(**a**) XRD pattern of unirradiated, Ni$^{+}$-beam-irradiated, and H$^{+}$-beam-irradiated CI powders. The inset shows the SEM images of the size and surface morphology of the unirradiated CI particles. XPS spectra of Fe 2p for (**b**) unirradiated, (**c**) Ni$^{+}$-beam-irradiated, and (**d**) H$^{+}$-beam-irradiated CI powders.

## Results and discussion

Figure 1a shows the X-ray diffraction (XRD) patterns for CI powders before and after Ni$^{+}$ and H$^{+}$ beam irradiation. Three main diffraction peaks correspond to single cubic $\alpha$-Fe (PDF#06-0696)^1,3,4,20^. In all the samples, no other peaks due to impurities are detected. The inset of Fig. 1 shows the scanning electron microscopy (SEM) image of the unirradiated CI, in which the size and morphology of the particles were observed. The particles are spherical with a smooth surface in shape, hindering the formation of spherical agglomerates^3,20^. It is thus expected that the uniform dispersion of CI powders in the matrix.

In Figs. 1b,c, the Fe 2p X-ray photoelectron spectrometry (XPS) spectra for CI powders before and after irradiation. The spectra were fitted well with four Gaussian lines after the Shirley background was subtracted using Fityk nonlinear curve fitting software^21^. The peaks at 711.3 eV and 725.3 eV were ascribed to the Fe$^{3+}$ 2p$_{2/3}$ and Fe$^{3+}$ 2p$_{1/2}$ electrons, respectively^4,5,12^. A satellite peak at $\sim$ 716 eV of the main Fe$^{3+}$ 2p$_{2/3}$ peak arises from Fe$_{2}$O$_{3}$ oxide that is shown in all the samples. The binding energy at 707 eV would correspond to the metallic Fe state^4,12^, but it is not seen in our data. Taking into account the presence of O 1s peak in the survey spectra for all the samples, we may suggest that some Fe elements on the surface of the samples before and after irradiation are oxidized to Fe$_{2}$O$_{3}$. Using the SRIM2008 package, we simulated the mean implantation depth and profile of Ni$^{+}$ beam with 140 keV and H$^{+}$ beam with 200 keV onto CI. The estimated depths for Ni$^{+}$ and H$^{+}$ beam were about 46 ± 21 nm and 860 ± 98 nm, respectively. From the inductive coupled plasma mass spectroscopy (ICP-MS) elemental analysis, we clearly observed $^{58}$Ni as much as 3.8 ppb for the irradiated particles, whereas it is below the detection limit for the unirradiated particles.

**Figure 2:**
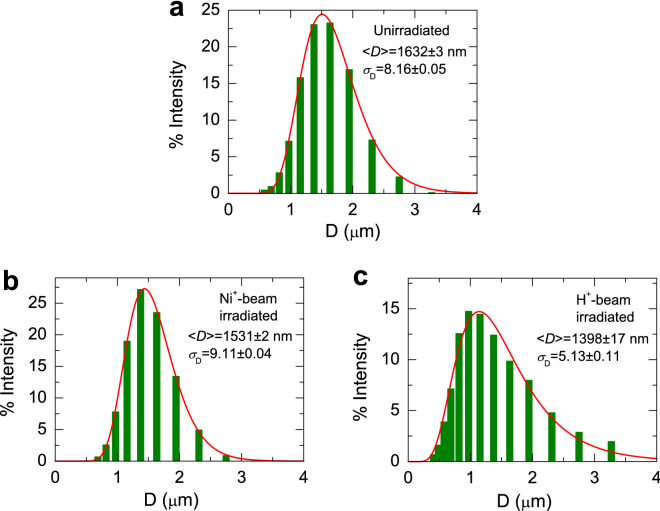
Particle size histograms (bars) obtained from the DLS analysis for (**a**) unirradiated, (**b**) Ni$^{+}$-beam-irradiated, and (**c**) H$^{+}$-beam-irradiated CI powders, and the fits of the data according to a log-normal distribution. The fits give the median particle diameter $\langle D \rangle$ = 1.632 ± 0.003 $\upmu$m and the distribution width $\sigma _{D}$ = 8.16 ± 0.05 for the unirradiated sample, $\langle D \rangle$ = 1.531 ± 0.002 $\upmu$m, $\sigma _{D}$ = 9.11 ± 0.04 for Ni$^{+}$-beam-irradiated sample , and $\langle D \rangle$ = 1.398 ± 0.017 $\upmu$m, $\sigma _{D}$ = 5.13 ± 0.11 for the H$^{+}$-beam-irradiated sample.

Figure 2 shows the particle size histogram obtained from the dynamic light scattering (DLS) measurement, together with fits by using a log-normal distribution of particle diameters. The distribution may be given by^22,23^

1 $$\begin{aligned} f(D)=\frac{1}{\sqrt{2\pi }\sigma D} \mathrm {exp}\left( -\frac{\mathrm {ln}^{2}\left( \frac{D}{\langle D \rangle }\right) }{2 \sigma _{D}^{2}}\right) , \end{aligned}$$

where ${\langle D \rangle }$ and $\sigma _{D}$ denote the median particle diameter and the distribution width, respectively. The distribution may be fitted with a single log-normal distribution in all the samples, as shown in Fig. 2. Before and after beam irradiation, the distribution of the particles is thus a single distribution with similar ${\langle D \rangle }$. It is in agreement with similar linewidths ($\sim$ 0.91$^{\circ }$ for the peak at (110)) in XRD peaks of the samples, indicative of similar crystallite sizes according to the Debye–Scherrer formula^4^. Hence, it should be noted that the size and the shape of the particles do not be a cause of change in magnetic properties, due to their consistency regardless of beam irradiation.

**Figure 3:**
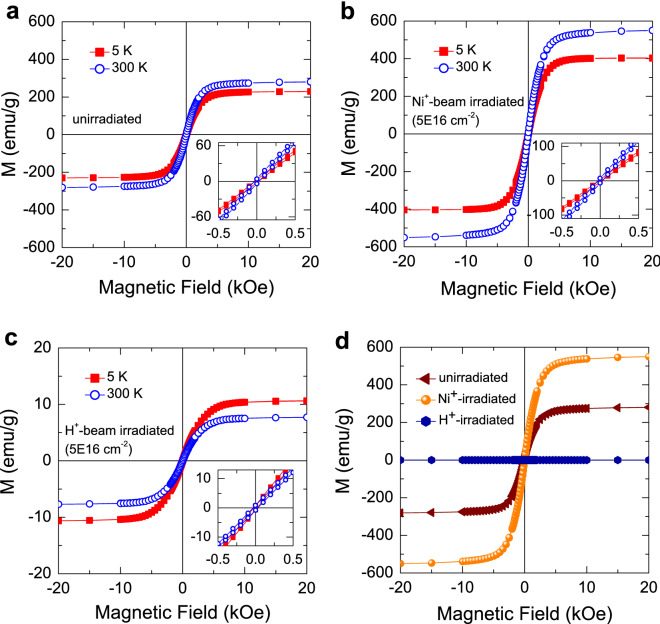
Magnetization curves of (**a**) unirradiated, (**b**) Ni$^{+}$-beam-irradiated, and (**c**) H$^{+}$-beam-irradiated CI powders measured at 300 K and 5 K by using SQUID. The inset of each figure shows the curves measured at the lower magnetic field. (**d**) Magnetization curves of unirradiated, Ni$^{+}$-beam-irradiated and, H$^{+}$-beam-irradiated CI powders measured at 300 K for comparison.

Figure 3a–c show magnetization curves of the CI particles before and after Ni$^{+}$- or H$^{+}$-beam irradiation measured at 5 K and 300 K, in which the magnetic field swept from − 20 and 20 kOe. As shown in the figure, all the samples show the magnetic hysteresis curves with very small similar coercive fields of $H_{\text {c}}$ = $\sim$ 21 Oe and $H_{\text {c}}$ = $\sim$ 29 Oe at 5 K and 300 K, respectively. Intriguingly, the magnetization is greater at a high temperature of 300 K than at a low temperature of 5 K in the unirradiated and Ni$^{+}$-beam-irradiated samples, whereas the reverse is true for the H$^{+}$-beam-irradiated sample. Note that the saturation magnetization $M_{\text {s}}$ of the Ni$^{+}$ irradiated sample is about two times greater than that of the unirradiated sample, indicating Ni$^{+}$ ions doped into CI particles may enhance the magnetization. In contrast, H$^{+}$ ions doped into CI particles exhibit a decrease in $M_{\text {s}}$ at 5 K and 300 K. Figure 3d shows magnetization curves comparing the samples before and after irradiation measured at 300 K. It is clearly observed that the $M_{\text {s}}$ of Ni-doped sample is about two times greater than that of the unirradiated sample, whereas that of H-doped sample exhibits a remarkable decrease.

Typically, in superparamagnetic nanoparticles, the magnetic anisotropy on the surface may increase, but in the bulk they may have the ferromagnetic single domain^24–26^. A superparamagnetic behavior shows an increasing magnetization with increasing applied magnetic field with no remanent magnetization and coercive field in the nanoparticles. However, in our hysteresis curves, the $M_{\text {s}}$ was observed at $\sim$ 10 kOe, which is unlikely to the superparamagnetic behavior. Furthermore, the $H_{c}$ should become to be zero, if the system exhibits a superparamagnetic behavior, which is thus not our case. Based on our XPS data in Fig. 1b,c, Fe$_{2}$O$_{3}$ phase on the CI particles was obviously observed, from which the canting of the surface spins or weak ferromagnetism can be introduced upon increasing temperature. $\alpha$-Fe$_{2}$O$_{3}$ is a typical weak ferromagnet, exhibiting stronger ferromagnetic behavior at higher temperature^14,15^. Previously, in CI and soft composites, ferromagnetic properties are observed^7,12^. However, in contrast to our data, the $M_{\text {s}}$ exhibited an increase with decreasing temperature, presumably due to an increase of RKKY interactions. We thus suggest that the anomalous increase of magnetization with increasing temperature may be attributed to the magnetic properties from Fe$_{2}$O$_{3}$ phase.

**Figure 4:**
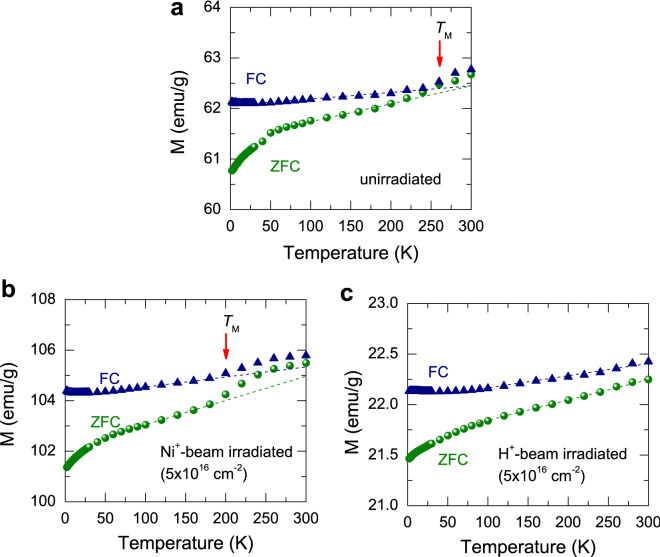
ZFC-FC magnetization curves of (**a**) unirradiated, (**b**) Ni$^{+}$-beam-irradiated, and (**c**) H$^{+}$-beam-irradiated CI powders measured with an applied magnetic field of 500 Oe. The dashed lines denote a linear behavior of the magnetization as a guide to the eyes.

To get a better understanding of the magnetic behavior, we measured the magnetization as a function of temperature. In Fig. 4, the zero-field cooled (ZFC) and field cooled (FC) magnetization curves are displayed. First, we may consider the ZFC curves for both samples. When the particles cooled in zero field, each particle aligns with the random direction of the easy axis due to its magnetic anisotropy energy barrier being enhanced with decreasing temperature. Because of the random orientation of the easy axis, the net magnetization approaches zero at very low temperatures^24,27,28^. Then, as the particles are warmed up to 300 K in the presence of the field (500 Oe), they can switch their magnetization direction from the easy axis to the applied field due to thermal energy, leading to an increase in the overall magnetization, as shown in the ZFC curves of Fig. 4. For the FC curves, upon decreasing temperatures under an applied field (500 Oe), the direction of the magnetic moment for each particle will tend to align with the easy axis closest to the applied field direction, and thus remain locked in that direction. In other words, the magnetic anisotropy energy barrier increases with reducing the temperature, making the magnetic moments align with the direction of the easy axis.

In the unirradiated sample in Fig. 4a, the magnetizations of ZFC and FC curves exhibit a more increment above $\sim$ 260 K, deviating from a linear behavior as noted by the dashed lines. This behavior is also shown in the Ni$^{+}$-beam-irradiated sample, however, deviating greater at a lower temperature of $\sim$ 200 K. This deviation in magnetization implies a transition in magnetic interaction, as is the case in $\alpha$-Fe$_{2}$O$_{3}$ known as undergoing a Morin transition at $T_{\text {M}}$ $\sim$ 260 K^14,29^. The spin reorientation (Morin) transition implies that a system undergoes from a canted antiferromagnet, i.e., weak ferromagnet above $T_{\text {M}}$ to no canting state, i.e., pure antiferromagnet below $T_{\text {M}}$, arising from a change in an anisotropic exchange interaction between the in-plane and out-of-plane states^16–18^. Although the transition temperature $T_{\text {M}}$ is exactly the same as $\alpha$-Fe$_{2}$O$_{3}$^14,17–19,30^, the transition in our CI is significantly suppressed compared to that of $\alpha$-Fe$_{2}$O$_{3}$. However, in Fig. 4b, a greater deviation in magnetization at lower $T_{\text {M}}$ was observed and may be associated with the greater $M_{\text {s}}$ for the Ni$^{+}$-beam-irradiated sample, as shown in Fig. 3b. By Ni$^{+}$ ion beam irradiation, the Ni ions are doped onto the surface of the particles (depth $\sim$ 46 nm) as previously mentioned, thereby giving rise to a greater spin reorientation.

We now discuss how Ni$^{+}$ doping into $\alpha$-Fe$_{2}$O$_{3}$ on our CI particles enhances magnetization and induces a steeper transition at the lower temperature. We note that $\alpha$-Fe$_{2}$O$_{3}$ is an insulator with a bulk DM interaction causing a weak macroscopic in-plane canting, whereas CI powder is a ferromagnetic material with high $M_{\text {s}}$ and low $H_{\text {c}}$, and thus no DM interaction resulting in a spin orientation^4,17,18^. Consequently, our magnetic behavior undergoing the Morin transition may arise from the surface of CI particles coated with $\alpha$-Fe$_{2}$O$_{3}$. Surface modification by Ni doping may enhance overall saturation magnetization and induces a steeper transition at the lower temperature of $\sim$ 200 K. In contrast, by H doping the CI powders exhibit no Morin transition (Fig. 4c), and a decrease in overall magnetization (Fig. 3c,d). We suggest that Ni$^{+}$ ion beam may increase uncompensated surface spins, whereas H$^{+}$ ion beam with no electron may decrease the surface spins. Comparably, Bahuguna et al.^14^ recently found that surface modification in $\alpha$-Fe$_{2}$O$_{3}$ may induce a significant improvement in magnetic properties. They explain that the enhancement in magnetization may be attributed to the electrophilic fluorination, giving rise to uncompensated spins, in agreement with our picture.

## Conclusions

In summary, we have investigated the magnetism in the CI powders before and after the Ni$^{+}$ and H$^{+}$ ion beam irradiation. We have found that the introduced $\alpha$-Fe$_{2}$O$_{3}$ phase on the CI particles as confirmed by XPS spectroscopy leads to the anomalous enhanced magnetization at the high temperature, due to the Morin transition causing canted antiferromagnetism above the Morin temperature. In Ni$^{+}$-beam irradiated sample, ferromagnetic hysteresis curves were observed as in the unirradiated sample, but with substantially increased saturation magnetization with a steeper Morin transition behavior at the lower temperature. On the other hand, H$^{+}$ beam irradiation markedly suppresses the magnetization and makes the anomalous magnetization reverse disappear, concomitant with the absence of the Morin transition. It reveals that the beam irradiation does not affect the size and the shape of the particles, thus not causing the change in magnetic properties. The irradiation of Ni$^{+}$ beam with electrons may provide the uncompensated surface spins on the CI particles, thereby enhancing magnetization and the Morin transition, but H$^{+}$ beam with no electron may suppress the magnetization and the Morin transition.

## Methods

### Sample preparation and characterization

Pure CI powder (grade EW; BASF, Germany) was used as-received and consists of particles with a diameter of 0.4–3.3 $\upmu$m. The crystalline phase structure of CI powders before and after irradiation was examined by X-ray diffraction (XRD) by using a MiniFlex 600 (RIGAKU, Japan) diffractometer with 3-kW monochromatic Co radiation ($\lambda$ = 1.79 $\AA$) in the range 5$^{\circ }$–90$^{\circ }$ 2$\theta$ of diffraction angle. The X-ray photoemission spectroscopy (XPS) measurements were performed by using a Shimadzu ESCALAB250 system for the surface analysis of the CI particles. Size and morphology of the particles were examined using a scanning electron microscopy (SEM) (JEOL, Tokyo, Japan). The distribution of the particle sizes of the CI powders before and after irradiation was examined by a dynamic light scattering (DLS) analyzer (Nanotrac wave). Inductive coupled plasma mass spectroscopy (ICP-MS) (Perkin Elmer, NexION 350D) was carried out to measure the element concentration of Ni in CI powder.

### Ion-beam irradiation

To irradiate the Ni$^{+}$ ion beam onto the samples, we used a metal ion accelerator with the beam energy of 140 keV. For H$^{+}$ beam irradiation, we used a gas ion accelerator with the energy of 200 keV. The irradiated doses of Ni$^{+}$ and H$^{+}$ beam were equivalently 5 $\times$ 10$^{16}$ cm$^{-2}$. During the irradiation, the sample was kept at room temperature using a cooling system. The samples were dispersed using 2-butoxyethyl acetate and then drop-casted onto SiO$_{2}$, followed by drying at 373 K using a drying oven for 2 h. Consequently, the $\sim$ 100-$\upmu$m-thick CI films were irradiated by the beam. To enhance homogeneity for beam irradiation, all the particles on the irradiated samples were again blended and then prepared as the films undergoing the same preparation process to irradiate the beam once again. To estimate the Ni$^{+}$ and H$^{+}$ penetration depths, we use the SRIM 2008 code^31^.

### Temperature- and field-dependent magnetometry

The magnetic characterization were performed using a superconducting quantum interference device (SQUID) magnetometer of Quantum Design MPMS at Korea Basic Science Institute (KBSI). $M(\text {T})$ curves were recorded from 2 to 300 K under 500 Oe applied field and under zero-field. $M(\text {H})$ curves were made in the field range of ± 70 kOe at temperatures of 5 K and 300 K.
